# Supplementary material for: Resolving the full spectrum of human genome variation using Linked-Reads
Source: Genome Res. 2019 Apr;29(4):635–45. doi: 10.1101/gr.234443.118 (PMC6442396; doi:10.1101/gr.234443.118)
Supplement: Supplemental Material [file supp_gr.234443.118_Supplemental_longranger-2.2.zip › longranger-2.2.tar.bz2!/lr2.2-public/lib/stan/cmdstan/stan/doc/api/html/initialize__state_8hpp.html]

Stan: src/stan/common/initialize\_state.hpp File Reference


|  |  |
| --- | --- |
|  | Stan  2.5.0  probability, sampling & optimization |


- Main Page
- Related Pages
- Namespaces
- Classes
- Files

- File List
- File Members

All Classes Namespaces Files Functions Variables Typedefs Enumerations Enumerator Friends Macros Pages

- src
- stan
- common

Namespaces |
Functions

initialize\_state.hpp File Reference

`#include <string>`  
`#include <iostream>`  
`#include <math.h>`  
`#include <stan/math/matrix/Eigen.hpp>`  
`#include <boost/lexical_cast.hpp>`  
`#include <boost/random/additive_combine.hpp>`  
`#include <boost/random/uniform_real_distribution.hpp>`  
`#include <boost/random/variate_generator.hpp>`  
`#include <stan/model/util.hpp>`  
`#include <stan/gm/error_codes.hpp>`  
`#include <stan/common/context_factory.hpp>`  
`#include <stan/common/write_error_msg.hpp>`

Go to the source code of this file.

|  |  |
| --- | --- |
| Namespaces | |
|  | stan |
|  | Probability, optimization and sampling library. |
|  | |
|  | stan::common |
|  | |

|  |  |
| --- | --- |
| Functions | |
| template<class Model > | |
| bool | stan::common::initialize\_state\_zero (Eigen::VectorXd &cont\_params, Model &model, std::ostream \*output) |
|  | Sets initial state to zero. More... |
|  | |
| template<class Model , class RNG > | |
| bool | stan::common::initialize\_state\_random (const double R, Eigen::VectorXd &cont\_params, Model &model, RNG &base\_rng, std::ostream \*output) |
|  | Initializes state to random uniform values within range. More... |
|  | |
| template<class ContextFactory , class Model , class RNG > | |
| bool | stan::common::initialize\_state\_source (const std::string source, Eigen::VectorXd &cont\_params, Model &model, RNG &base\_rng, std::ostream \*output, ContextFactory &context\_factory) |
|  | Creates the initial state using the source parameter. More... |
|  | |
| bool | stan::common::get\_double\_from\_string (const std::string &s, double &val) |
|  | Converts string to double. More... |
|  | |
| template<class ContextFactory , class Model , class RNG > | |
| bool | stan::common::initialize\_state (const std::string init, Eigen::VectorXd &cont\_params, Model &model, RNG &base\_rng, std::ostream \*output, ContextFactory &context\_factory) |
|  | Creates the initial state. More... |
|  | |


---

[ Stan Home Page ]

*© 2011–2014,
Stan Development Team.*
